# Clinical experience with the Bicarbon heart valve prosthesis

**DOI:** 10.1186/1749-8090-2-8

**Published:** 2007-01-25

**Authors:** Yoshio Misawa, Tsutomu Saito, Hiroaki Konishi, Shin-ichi Oki, Yuichiro Kaminishi, Yasuhito Sakano, Hideki Morita, Kei Aizawa

**Affiliations:** 1 Division of Cardiovascular Surgery, Jichi Medical University, 3311-1 Yakushiji, Shimotsuke, Tochigi, 329-0498, JAPAN

## Abstract

**Bacground:**

We have previously reported mid-term results of a study, which ended in January 2000, on the Bicarbon valve. The study concluded that the valve showed excellent clinical results, associated with a low incidence of valve-related complications. In the present study, the same patients were prospectively followed for an additional 5 years.

**Methods:**

Forty-four patients had aortic valve replacement (AVR), 48 had mitral valve replacement (MVR), and 13 had both aortic and mitral valve replacement (DVR). The mean age of the 105 patients was 61.2 ± 11.3 years. The mean follow-up was 6.1 ± 1.9 years with a cumulative follow-up of 616 patient-years.

**Results:**

There were 5 early deaths (4.7%: 4 in the AVR group and 1 in the MVR group) and 21 late deaths (3.4%/patient-year: 5 valve related deaths and 16 valve unrelated deaths). Survival at 8 years was 75.2 ± 7.0% in the AVR group, 76.6 ± 6.2% in the MVR group, and 55.4 ± 16.1% in the DVR group. The linearized incidence of thrombo-embolic complications, hemorrhagic complications, and paravalvular leaks in all patients was 0.65 ± 1.48%, 0.81 ± 1.69%, and 0.16 ± 0.54%/patient-year respectively. No other complications were observed.

**Conclusion:**

The Bicarbon prosthetic heart valve has shown excellent long-term clinical results, associated with a low incidence of valve-related complications.

## Background

Both mechanical and bioprosthetic heart valves have become more durable and less thrombogenic, possessing excellent clinical outcomes and hemodynamic features. However, lifelong anticoagulant therapy is inevitable for patients with mechanical prosthetic valves, and those with bioprosthetic valves have higher risks of structural valve dysfunction than those with mechanical ones. In mechanical valves, bileaflet prosthetic heart valves are more preferably implanted than tilting disc valves, and surgeons choose some of them for valve replacement according to their own preference and the patients' informed consent. Many long-term clinical results showed excellent clinical performances of mechanical prostheses.

The Bicarbon valve (Sorin Biomedica Cardio, Saluggia, Italy) was introduced for clinical application at our institute in 1997 [1]. The purpose of this study is to evaluate prospective clinical performances of the Bicarbon valve implanted at a single center in Japan.

## Methods

Between February 1997 and December 2000, 107 patients were implanted with 120 Bicarbon valves at the Jichi Medical University Hospital. Two of the patients were excluded for the presence of two different types of valves, because they had been already implanted with another valve and received a Bicarbon valve in a different position. Forty-four patients had aortic valve replacement (AVR), 48 had mitral valve replacement (MVR), and 13 had both aortic and mitral valve replacement (DVR). There were 55 men and 50 women. Patients between the sixth and eighth decade consisted of the main candidates, and the mean age was 61.2 ± 11.3 years for all patients. The mid-term results of 105 patients with a Bicarbon valve as of the end of January 2000 have already been reported [2]. Clinical data of the same patients was evaluated on the basis of mortality and morbidity analysis until the end of December 2005.

At the operation, the myocardium was protected by moderate hypothermia, and by ante-grade with or without retro-grade intermittent perfusion of blood cardioplegic solution. For MVR, the posterior mitral valve apparatus was preserved, and an anti-anatomical position was chosen for implantation. Heparin sodium injection was our usual postoperative care during the early postoperative period, followed by oral anticoagulant therapy. Concomitant cardiac surgery procedures were tricuspid annulus repair in 28 patients, coronary artery bypass grafting in 16, maze procedure in 10, aortic root replacement in 6, and others [2]. Three patients with AVR and 7 with MVR had undergone valve surgery once or twice previously. Our postoperative anticoagulant therapy consisted of both warfarin potassium and anti-platelet agents such as dipyridamole or aspirin. The INR (international normalization ratio) jwas controlled to maintain values between 1.8 and 3.3 for patients having MVR and DVR, and was between 1.3 and 1.8 for AVR patients before 2000. Since then, INR was between 1.8 and 3.3 for all patients. For patients with atrial fibrillation who have mechanical valves, an INR of at least 2.5 is recommended. Thus, for young patients, our criterion is based on the recommendation, but for aged patients with some risk factors for cerebral bleeding, an INR is below the level of the recommendation [3].

Morbidity analysis included all cardiovascular complications as defined in Edmunds *et al*., Guidelines for Reporting Morbidity and Mortality after Cardiac Valve Operations [4]. Mortality data and incidence of clinical adverse events were analysed by means of the Kaplan-Meier actuarial method. The probability of freedom from the first occurrence of each complication was graphically represented with regard to the follow-up time. Each adverse event (death, thrombosis, embolism, anticoagulant related bleeding, endocarditis, non structural dysfunction) was summarised by means of linearized rates, calculated as the number of occurred events divided by the total number of patient-years (cumulative follow-up). This analysis was also conducted for re-operation events. For each linearized rate, upper confidence limits (95%) were provided, according to the method reported by Grunkemeier and Anderson [5]. All analyses were performed for the whole sample of data and stratified by implant site (aortic, mitral, and both aortic and mitral). For continuous variables descriptive statistics (mean, standard deviation, range) were provided.

As of the end of December 2005, 105 patients had participated in the follow-up study through an office interview, personal phone call, or mail interview. All patients were followed-up (100% completeness of follow-up) with a mean follow-up of 6.1 ± 1.9 years overall (AVR, 6.3 ± 1.8 years; MVR, 6.1 ± 1.9 years; DVR, 5.6 ± 2.2 years) and a cumulative follow-up of 616 years (257 years for AVR, 286 years for MVR, and 73 years for DVR).

## Results

1. Freedom from mortality and morbidity

a) Survival and clinical functional class

**Figure 1:**
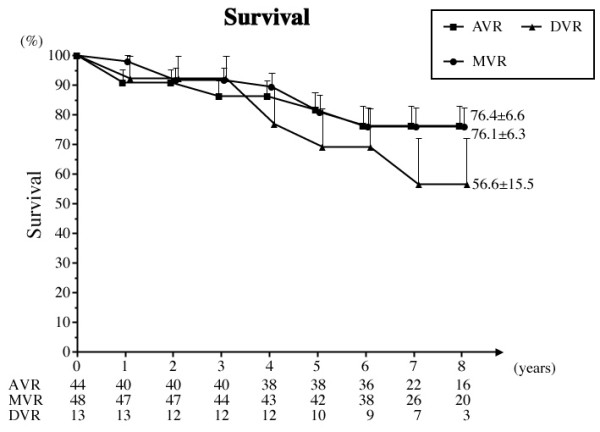
**Survival curve**. Numerical values of the graph express numbers of patients at the time of follow-up. Abbreviations; AVR: aortic valve replacement, MVR: mitral valve replacement, DVR: both aortic and mitral valve replacement.

Twenty-six patients died, including 5 hospital deaths (10 AVRs, 11 MVRs and 5 DVRs). One valve related death occurred in the AVR group, 2 in the MVR group and 2 in the DVR group. The survival rates at 8 years were 73.6 ± 4.5% overall, 75.2 ± 7.0% for AVR, 76.6 ± 6.2 % for MVR, and 55.4 ± 16.1% for DVR (Fig. 1). Preoperatively, 91.4% of the patients were in the New York Heart Association (NYHA) functional class III or IV, and 96.8% were in class I or II at follow-up.

b) Embolic events

**Figure 2:**
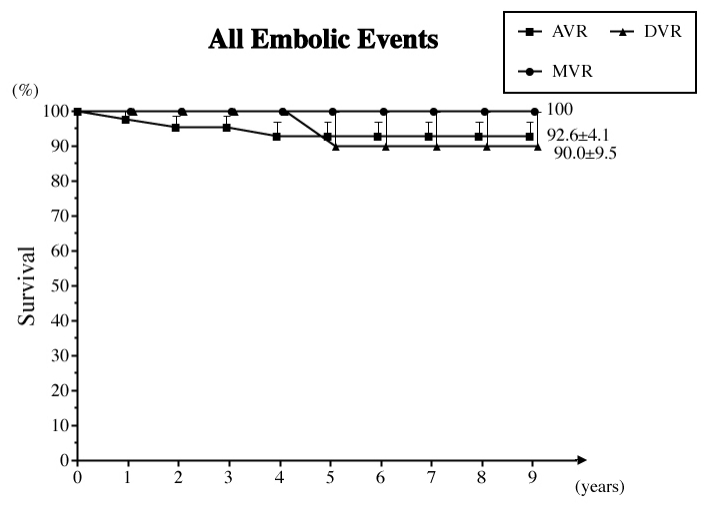
**Freedom from all embolic events**. Abbreviations; AVR: aortic valve replacement, MVR: mitral valve replacement, DVR: both aortic and mitral valve replacement.

Three embolic events occurred in the AVR group and 1 in the DVR group as follows: 1 non-obstructive mesenteric infarction, 1 transient ischemic cerebral accident and 2 strokes. The freedom from embolic episodes at 8 years was 95.8% overall, 93.6% in the AVR group, and 90.0% in the DVR group (Fig. 2).

c) Thrombosis

No thrombotic episodes occurred in this study.

d) Anticoagulant related bleeding

**Figure 3:**
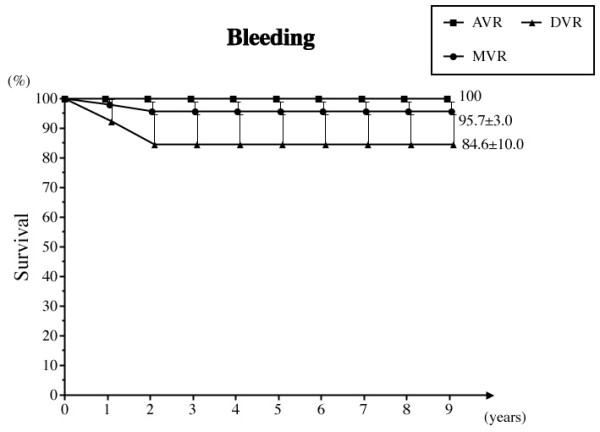
**Freedom from anticoagulant related bleeding**. Abbreviations; AVR: aortic valve replacement, MVR: mitral valve replacement, DVR: both aortic and mitral valve replacement.

Three bleeding episodes occurred in the MVR group; 2 for gastric hemorrhage and 1 for fatal cerebral hemorrhage. Two cerebral hemorrhage events (1 fatal) occurred in the DVR group. The freedom from anticoagulant related bleeding at 8 years was 96.0% overall, 95.6% in the MVR group, and 84.6% in the DVR group (Fig. 3).

e) Non-structural prosthetic valve dysfunction

**Figure 4:**
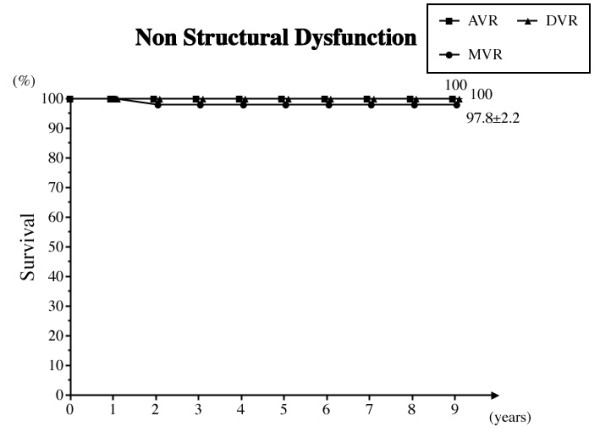
**Freedom from non-structural prosthetic valve dysfunction**. Abbreviations; AVR: aortic valve replacement, MVR: mitral valve replacement, DVR: both aortic and mitral valve replacement.

One occurrence of non-structural dysfunction in the MVR group for peri-prosthetic valvular leak required re-operation. The freedom from non-structural dysfunction at 8 years was 98.9% overall and 97.8% in the MVR group (Fig. 4).

f) Endocarditis

No endocarditis occurred.

g) Re-operation

One patient required re-operation for peri-prosthetic leak in the MVR group. The freedom from re-operation at 8 years was 98.9% overall and 97.8% in the MVR group.

h) Valve related death

**Figure 5:**
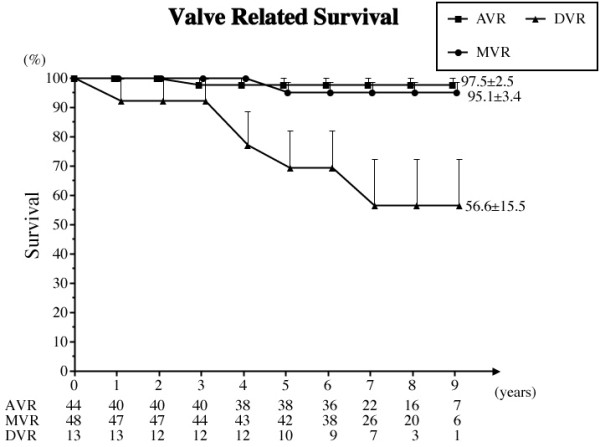
**Freedom from valve related death**. Abbreviations; AVR: aortic valve replacement, MVR: mitral valve replacement, DVR: both aortic and mitral valve replacement.

One valve related death occurred in the AVR group, 2 occurred in the MVR group and 2 occurred in the DVR group. The freedom from valve related death at 8 years was 94.6% overall, 97.4% in the AVR group, 95.1% in the MVR group, and 56.6% in the DVR group (Fig. 5).

2. Linearized rates and upper confidence rates of events (Table 1)

**Table 1:** Linearized Rates of Events

	OVERALL		AVR		MVR		DVR	
	%/pt-yr	95% CR	%/pt-yr	95% CR	%/pt-yr	95% CR	%/pt-yr	95% CR

All Embolic Events	0.65	1.48	1.16	3.01	0.00		1.37	7.10
Non Structural Dysfunction	0.16	0.84	0.00		0.35	1.81	0.00	
Bleeding	0.81	1.69	0.00		1.05	2.71	2.74	8.77
Valve Related Death	0.81	1.69	0.39	2.01	0.70	2.24	2.74	8.77

Abbreviations;

OVERALL: all patients including AVR, MVR, and DVR patients

AVR: aortic valve replacement, MVR: mitral valve replacement, DVR: both aortic and mitral valve replacement

Pt-yr: patient year, CR: confidential rate

The linearized rates and 95% confidence rates of each event overall were 0.65 ± 1.48%/patient-year for embolic events, 0.81 ± 1.69%/patient -year for bleeding, 0.16 ± 0.84%/patient year for non-structural dysfunction, and 0.81 ± 1.69%/patient-year for valve related death. The linearized and 95% confidence rates of the AVR group were 1.16 ± 3.01%/patient-year for embolic events and 0.39 ± 2.01%/patient-year for valve related deaths. The linearized and 95% confidence rates of the MVR group were 0.35 ± 1.81%/patient-year for non-structural dysfunction, 1.05 ± 2.71%/patient-year for bleeding, and those of the DVR group were 1.37 ± 7.10%/patient-year for thrombo-embolic events, and 2.74 ± 8.77%/patient-year both for bleeding and for valve related deaths.

## Discussion

No structural valve dysfunction has been observed in recently available mechanical heart valves. However, non-structural mechanical valve dysfunction, and thromboembolic, or hemorrhagic episodes, still lead to complications after valve surgery. Some patients need reoperation because of prosthetic valve dysfunction caused by restricting prosthetic valve function valve with thrombosis or pannus formation and peri-prosthetic valvular leaks with or without infective endocarditis [6,7].

Lowering thrombo-embolic episodes after implantation is the key for success when a mechanical valve is invented. The Bicarbon valve is composed of 2 leaflets convex toward the annulus. The 2 lateral orifices and the central orifice contribute to making the 3 blood streams through the prosthesis. The valve is designed on the basis that the blood flow through each of the 3 orifices is equal in amount, parallel, and laminar, preventing turbulent blood flow through the valve. Another important feature of the Bicarbon valve is the hinge rolling action that permits total flushing of the blood-exposed surfaces [8]. These structural features are supposed to lower thrombo-embolic episodes and hemolysis after implantation [9].

Excellent early and midterm clinical results of the Bicarbon valve have been previously reported [2,10-15]. The longer the follow-up periods are, the more reliable the clinical results are. Long-term follow-up studies of the valve are not enough to evaluate its clinical performance. With regard to single center mid-term results, Goldsmith and colleagues reported that the Bicarbon valve has a satisfactory clinical performance with low complication rates [14]. A multi-center study of the Bicarbon valve in Europe with a mean follow-up of 2.2 +/- 1.5 years showed 5% early deaths, and 4.4% late deaths [15]. This study also showed that survival of all the patients was 87% 5 years after the operation. The linearized incidence of valve thrombosis was between 0.06 and 0.69%/patient-years among the AVR, MVR and DVR groups, and that of embolic episodes was between 1.13 and 2.14%/patient-years. Bleeding complications occurred at the rate of 0.69 to 1.26%/patient-years.

Previous studies have shown that with long-term experience with the Bicarbon valve, freedom from valve thrombosis at 7 to 9 years was between 97 and 99.4%, that from embolic episodes was between 64 and 93%, and that from bleeding complications was between 82 and 98.6% [16-18]. Freedom from endocarditis and nonstructural valve dysfunction at 7 to 9 years was between 95 and 99% and between 84 and 98.7%, respectively. Actuarial analysis at 7 to 9 years showed an overall survival between 63.9 and 88%.

Long-term clinical experience with St. Jude Medical and Carbomedics bileaflet mechanical valves for AVR and MVR have shown that the rates of thrombosis were between 0.73 and 3.4%/patient-year, and that the 10-year freedom from thrombosis was between 77 and 94.2% [19-25]. In addition, the rates of bleeding were between 0.52 and 2.7%/patient-year, and the 10-year freedom from bleeding was between 77 and 96.4%. This means that these bileaflet mechanical valves work well without different clinical performances.

We reported our echocardiographic evaluation and mid-term clinical experience with the Bicarbon bileaflet prosthetic heart valve in 1999 and 2002. The echocardiographic study showed that 3 blood streams through the prosthesis were equal in amount, and parallel with similar flow velocity, indicating the hemodynamic superiority of the prosthesis to other bileaflet valves [1]. The latter clinical follow-up report described the clinical results of survival, and valve-related morbidity or mortality on the basis of the valve, and concluded that similar results were obtained to those of other mechanical valves associated with a low incidence of valve-related morbidity and mortality [2].

The present follow-up study gives additional evidence of low rates of valve-related complications of the Bicarbon valve, showing that the 8-year freedom from thrombo-embolism was 93.6% in the AVR and 100% in the MVR groups, and the 8-year freedom from bleeding was 100% in the AVR and 95.6% in the MVR groups. The rate of thrombo-embolism in the AVR group was 1.16%/patient-year, and that of bleeding in the MVR group was 1.05%/patient-year. Thrombo-embolic complications occurred in patients of the AVR and DVR groups, whose anticoagulant therapy was well controlled. All patients received both warfarin potassium and anti-platelet agents. An INR between 1.3 and 1.8 was our standard for patients who had AVR before 2000. We have previously reported that AVR patients tended to show higher incidences of lethal hemorrhagic complications [26]. Thus, INR for these patients was lower than INR for patients who had MVR or DVR. However, our previous study implies that INR should be maintained between 1.8 and 3.3 for all patients having mechanical valve replacement [2]. Hemorrhagic complications occurred in 5 patients with MVR or DVR, including 2 deceased cases because of cerebral bleeding. Anticoagulant therapy was well controlled in these 2 cases. This might indicate an adverse influence of anticoagulant therapy on the patients' prognosis in cases of complications. Therefore, we believe that not only careful anticoagulant therapy but adequate control of risk factors such as hypertension and diabetes mellitus is required to prevent lethal complications. Non-structural prosthetic valve dysfunction due to peri-valvular leak was observed in only one MVR patient. Operative findings showed a small fistula, 3 mm in diameter, between the left ventricle and atrium, which was located near the posterior fibrous trigon. The patient had no apparent history of infective endocarditis after initial valve replacement. Postoperative NYHA clinical functional improvement was satisfactory in all groups. Low cardiac output syndrome led to lethal complications in those patients with advanced stage IV NYHA classification. In order to obtain better clinical outcomes, an operation before advanced clinical function should be considered, and more intensive peri-operative management may be mandatory for patients with far advanced heart failure.

## Conclusion

We analyzed the cases of 105 patients who received a Bicarbon prosthetic heart valve implantation at the mitral and/or aortic position. This long-term single center study with a Bicarbon prosthetic heart valve shows excellent clinical results associated with a low incidence of valve-related mortality and morbidity.
